# Genetic continuity across a deeply divergent linguistic contact zone in North Maluku, Indonesia

**DOI:** 10.1186/1471-2156-12-100

**Published:** 2011-11-18

**Authors:** Jason A Wilder, Murray P Cox, Andrew M Paquette, Regan Alford, Ari W Satyagraha, Alida Harahap, Herawati Sudoyo

**Affiliations:** 1 Department of Biological Sciences, Northern Arizona University, Flagstaff, Arizona, USA; 2 Institute of Molecular Biosciences, Massey University, Palmerston North, New Zealand; 3 Eijkman Institute for Molecular Biology, Jakarta, Indonesia

## Abstract

**Background:**

The islands of North Maluku, Indonesia occupy a central position in the major prehistoric dispersal streams that shaped the peoples of Island Southeast Asia and the Pacific. Within this region a linguistic contact zone exists where speakers of Papuan and Austronesian languages reside in close proximity. Here we use population genetic data to assess the extent to which North Maluku populations experienced admixture of Asian genetic material, and whether linguistic boundaries reflect genetic differentiation today.

**Results:**

Autosomal and X-linked markers reveal overall Asian admixture of 67% in North Maluku, demonstrating a substantial contribution of genetic material into the region from Asia. We observe no evidence of population structure associated with ethnicity or language affiliation.

**Conclusions:**

Our data support a model of widespread Asian admixture in North Maluku, likely mediated by the expansion of Austronesian-speaking peoples into the region during the mid Holocene. In North Maluku there is no genetic differentiation in terms of Austronesian- versus Papuan-speakers, suggesting extensive gene flow across linguistic boundaries. In a regional context, our results illuminate a major genetic divide at the Molucca Sea, between the islands of Sulawesi and North Maluku. West of this divide, populations exhibit predominantly Asian ancestry, with very little contribution of Papuan genetic material. East of the Molucca Sea, populations show diminished rates of Asian admixture and substantial persistence of Papuan genetic diversity.

## Background

The islands of North Maluku, Indonesia (also known as the Moluccas or Spice Islands) lie in a pivotal position on the migration routes of humans through the Indo-Pacific. Two major prehistoric movements of people are particularly important with respect to the linguistic, cultural, and genetic diversity of the region. First, the initial Pleistocene colonization of the western Pacific flowed from west to east through Indonesia to New Guinea and Australia, bringing the ancestors of modern Papuans and Australians into the region. Although the exact route(s) of this migration are not known, North Maluku lies along a probable pathway of inter-island travel between Sulawesi and New Guinea [1]. Indeed, archaeological evidence confirms human habitation in North Maluku as far back as 32,500 years before present [2]. The second major migration event with a significant impact on North Maluku was the spread of Asian Austronesian-speaking populations from the north via the Philippines into island Southeast Asia and Oceania. The oldest pottery found in North Maluku and associated with the Austronesian culture dates to 3,500 years before present [2]. Several linguistic and cultural aspects of the Austronesian expansion appear to have originated in the islands of North Maluku, suggesting it played an important role in the spread of Austronesians throughout the region [3]. As such, the prehistoric contributions to present-day communities in this region can largely be framed around these two main events.

**Figure 1:**
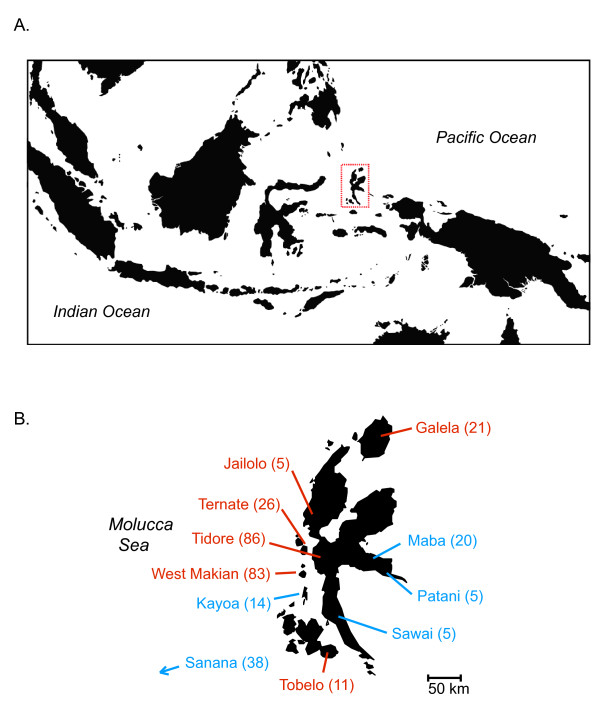
**Study Area**. A. Overview map of Island Southeast Asia. The region of North Maluku, Indonesia where the present study was performed is highlighted by the red dashed box. B. Focal study region showing the main island of North Maluku, Halmahera, and its satellite islands. Sampled populations are named together with numbers of sampled individuals in parentheses; red indicates speakers of Papuan languages, blue indicates Austronesian speakers. Map positions indicate general vicinities where individuals of sampled ethnicities reside (from [5]). The Sanana population lives on the island of Sanana, part of North Maluku province, approximately 80 km to the southwest of the region shown.

With respect to these prehistoric influences, North Maluku is particularly remarkable because it is one of only a handful of island groups in eastern Indonesia that maintains extant languages that likely reflect both the initial Pleistocene colonization event and subsequent immigration of Austronesians. Geographically, the most widespread languages in North Maluku are of Papuan origin and fall within the West Papuan language family; this family ultimately traces its ancestry back to the original Pleistocene colonization of Island Southeast Asia [4]. The West Papuan language group is particularly diverse on the Bird's Head peninsula of New Guinea, and North Maluku represents its western-most modern-day occurrence [5]. In addition to these Papuan languages there are also a number of Austronesian languages established in the area [6], particularly in the southern portions of the island of Halmahera and its satellite islands (Figure 1). As such, speakers of deeply divergent Papuan and Austronesian languages live in close proximity in this region today.

The mechanism by which Austronesian languages became established in North Maluku remains unknown. The diversity of Papuan languages in the area suggests that they were well-established prior to the arrival of Austronesians [2,7]. Two simple models of the establishment of Austronesian languages that make opposing genetic predictions are a Replacement Model and an Adoption Model. The first of these posits that Austronesian-speakers partially colonized North Maluku, displacing speakers of Papuan languages in the process. Even if there was some intermarriage between Papuan- and Austronesian-speakers, this model suggests that populations representing these two language groups will be genetically distinct from one another [8]. Conversely, the Adoption Model posits that speakers of Papuan languages in North Maluku adopted Austronesian languages through cultural contact with Austronesian speakers, not intermarriage. This model predicts no major change in the genetic composition of the resident pre-existing population [8,9], thereby proposing that there will be a minimal signature of Austronesian admixture in North Maluku and little genetic differentiation between Papuan and Austronesian-speaking populations. A genetic pattern consistent with the Adoption Model has been observed in many Austronesian-speaking populations in eastern Indonesia and Melanesia, and has been interpreted as evidence of widespread language borrowing in this region [10]. Of course, the Replacement and Adoption Models reflect opposite extremes on a spectrum of more complex scenarios.

Given the remarkable linguistic diversity of North Maluku, and the obvious importance of its geographic position for both Pleistocene and Austronesian migration, we have surprisingly little knowledge of the genetic composition of populations living in this region today. Because languages in North Maluku have both Asian and Papuan origins, our goal here is to test whether there is a genetic signature that also reflects this division.

One approach to this question would be to genotype large numbers (*i.e.*, hundreds of thousands) of single nucleotide polymorphisms (SNPs) across a range of samples (*e.g.*, using a SNP chip). However, from the perspective of information content, this approach is expensive. The vast majority of SNPs in a population are unlikely to show frequency differences between Asian and Papuan groups, and therefore have no statistical power to infer Asian-Papuan ancestry. Instead, information about ancestry proportions is carried by a relatively small number of SNPs with high frequency differences between the parental groups. Such SNPs are called ancestry informative markers (AIMs). AIMs are considerably cheaper to screen than genotyping with standard SNP chips, but they retain extremely high power to infer ancestry proportions. One key limitation is that AIMs only have statistical power to answer the questions they were designed to address - in this case, proportions of Asian versus Papuan ancestry. Nevertheless, this is the main axis of population history in the Indo-Pacific region today.

The research presented here genotypes 27 previously identified AIMs in 340 individuals representing 11 different ethnicities in North Maluku (Figure 1). This sampling scheme includes multiple populations speaking both Papuan and Austronesian languages. The study has two main aims: 1) to estimate the amount of Asian admixture among populations in the region; and 2) to test whether linguistically differentiated groups are also genetically distinct from one another. Addressing these questions will enhance our understanding of how the remarkable contact zone in North Maluku originally formed, as well as shed light on the dynamics of the expansion of Austronesian speakers through the region.

## Results

### Asian Admixture in North Maluku

To detect the genetic signature of Asian admixture into North Maluku, we genotyped 11 autosomal and 16 X-chromosomal AIMs in a sample of approximately 340 individuals. These unlinked and statistically independent markers were chosen because they have alleles that are highly differentiated between Asian and Melanesian source populations and are therefore useful to detect contributions from each of these potential parental sources [11]. The source populations used to identify these AIMs were the Southern Han Chinese and highland Papua New Guineans. Other potential source populations have similar allele frequencies at these AIMs, and the choice of parental population has little effect on the power to detect admixture along this Asian-Papuan axis [11]. Because we typed numerous markers from both the X chromosome and the autosomes, we are also able to explore potential sex biases in rates of admixture.

Each of the AIMs included in this study exhibited alleles of intermediate frequency in our total sample from North Maluku, thus suggesting strong Papuan *and *Asian contributions to genetic diversity in this region (Additional File 1). Using a least squares-based estimator (see Methods section), the overall average estimate of Asian admixture is 0.72 for X chromosomal loci and 0.64 for autosomal loci (Table 1). This trend of higher X chromosomal than autosomal Asian admixture is observed for all individual populations except Kayoa and Sawai, but is only statistically significant for around half of the studied populations (*i.e.*, the point estimate of Asian admixture for X chromosomal loci exceeds the 95% confidence interval for autosomal loci). Nevertheless, the general pattern is indicative of female-biased gene flow into the region from Asia, as is consistent with earlier work [11]. There is very little heterogeneity among populations with respect to admixture estimates (Table 1). With the exception of the X chromosome data from Ternate, no outliers were observed after applying a 5% family-wise false discovery rate (FDR) correction for multiple testing.

**Table 1:** Asian admixture estimates

	Autosomes			X chromosome			All Loci Combined		
			
Population	N	Estimate	95% C.I.	N	Estimate	95% C.I.	N	Estimate	95% C.I.
**All Papuan**	**473.3**	**0.64**	**0.61 - 0.68**	**400.0**	**0.70**	**0.66 - 0.74**	**429.9**	**0.67**	**0.65 - 0.70**
Galela	42	0.65	0.58 - 0.73	30.8	0.76	0.69 - 0.84	35.3	0.72	0.66 - 0.77
Jailolo	10	0.52	0.38 - 0.67	7.9	0.83	0.66 - 1.00	8.8	0.72	0.58 - 1.00
Makian	165.6	0.65	0.61 - 0.69	130.6	0.70	0.65 - 0.75	144.9	0.68	0.64 - 0.71
Ternate	51.8	0.64	0.58 - 0.70	45.6	0.78	0.71 - 0.85	48.1	0.72	0.67 - 0.77
Tidore	171.3	0.67	0.63 - 0.71	152	0.67	0.62 - 0.72	159.9	0.67	0.64 - 0.70
Tobelo	22	0.69	0.60 - 0.78	15.8	0.76	0.65 - 0.87	18.3	0.73	0.65 - 0.81
**All Austronesian**	**201.1**	**0.62**	**0.58 - 0.65**	**166.2**	**0.70**	**0.66 - 0.75**	**180.4**	**0.66**	**0.64 - 0.70**
Kayoa	28	0.72	0.63 - 0.80	24.9	0.67	0.58 - 0.76	26.1	0.69	0.63 - 0.76
Maba	39.8	0.65	0.57 - 0.72	32.9	0.70	0.62 - 0.80	35.7	0.68	0.62 - 0.74
Patani	10	0.65	0.50 - 0.80	7.9	0.79	0.62 - 0.97	8.8	0.74	0.61 - 0.89
Sanana	75.8	0.56	0.50 - 0.61	63.6	0.70	0.64 - 0.76	68.6	0.64	0.60 - 0.68
Sawai	10	0.63	0.48 - 0.80	9.9	0.60	0.40 - 0.84	9.9	0.62	0.48 - 0.79

N indicates the average number of chromosomes typed per population across all AIMs in each category (X-linked or autosomal). The 'All Papuan' and 'All Austronesian' categories include individuals from ethnicities that did not meet our minimum sample size requirement (5 individuals) to be included in the population-based analysis.

### Genetic Differentiation of North Maluku Populations

By partitioning the genetic variation in our dataset to separate components observed among major language groups, among populations within language groups, and within populations (*i.e.*, performing an analysis of molecular variance, or AMOVA), we can begin to describe the genetic structure of human groups living in North Maluku. At both the X chromosome and autosomal loci, an average of ≥99.5% of the genetic variability can be found within populations (Table 2). The next largest component of variation exists among populations within language groups, and the smallest component falls among language groups.

**Table 2:** Hierarchical analysis of genetic differentiation (AMOVA)

	Among Language Groups	Among Populations, Within Groups	Within Populations
Autosomes	0.07%	0.43%	99.50%
X Chromosome	0.14%	0.33%	99.53%

Values indicate averages across all AIMs in each category.

Generally speaking, there was very little variability among markers in terms of the AMOVA results (Additional File 2). With one exception, the among-group and among-population components of variation for each AIM did not significantly exceed values expected by chance under panmixia. The sole exception was for X-linked marker rs5987967, which had an among-populations component of 5.19% (p < 0.0001); however, the among-group component for this marker was not significantly higher than expected by chance (and was, in fact, statistically indistinguishable from zero). These results suggest that for the X-linked and autosomal AIMs examined here, there is no meaningful among-group or among-population component of sampled genetic variation (*i.e.*, little genetic variation is apportioned according to linguistic groups or ethnicities).

**Figure 2:**
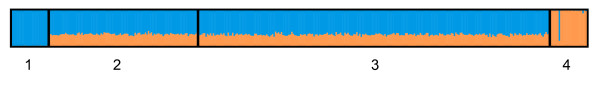
**STRUCTURE admixture plot**. STRUCTURE clearly delineates the two populations used to define the AIMs genotyped in the present study, the southern Han Chinese (1) and highland Papua New Guineans (4). Speakers of Austronesian and Papuan languages in North Maluku (groups 2 and 3, respectively) show a uniform degree of Asian admixture and no evidence of genetic differentiation. (Note the single individual with substantial Asian admixture in the highland Papua New Guinea sample).

Use of the clustering algorithm STRUCTURE also reveals little evidence for subdivision of language groups within North Maluku. When implementing analyses using STRUCTURE, we included our North Maluku dataset together with genotypes in the mainland Asian and Papuan populations from which the AIMs used here were originally developed. Given that our dataset was specifically designed to capture variation along the Asian-Papuan axis, we emphasize that only K = 2 has any natural interpretation in the context of the present study. Consistent with this, the estimated likelihood of our data plateaus at K = 2, suggesting this as the maximum number of latent groups necessary to capture the signal of genetic differentiation [12]. At K = 2 the results of the STRUCTURE analysis clearly show the Asian and Papuan parental populations to anchor the extremes of Asian-Papuan divergence, with our North Maluku samples showing evidence of extensive admixture between these two latent groups (Figure 2). There is no apparent differentiation of Austronesian- versus Papuan-speakers; the component of ancestry shared with Asia in the North Maluku population averages 0.67 (95% confidence interval; 0.62-0.72) among Austronesian-speakers and 0.68 (95% confidence interval; 0.62-0.74) among Papuan-speakers. It is notable that these admixture estimates are extremely close to those obtained with the least-squares method described above, and also show remarkably little variation among individuals or populations.

**Figure 3:**
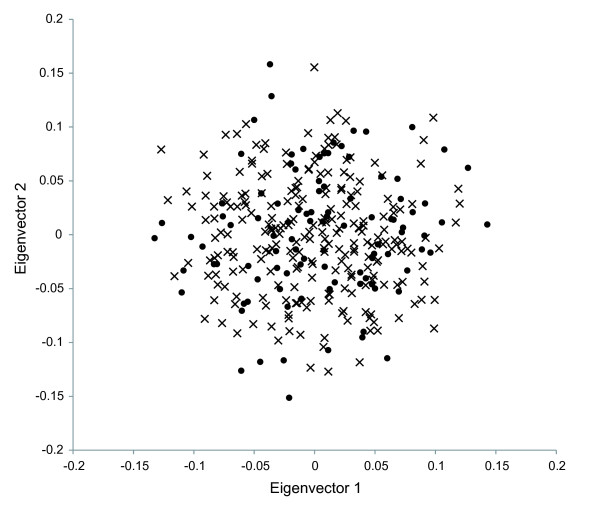
**PCA Plot**. The individual-based PCA reveals no significant difference in the distributions of speakers of Austronesian (indicated by black circles) and Papuan (indicated by 'x') languages along the two principal eigenvectors.

As a final analysis of differentiation between Austronesian- and Papuan-speakers, we performed an individual-based principal component analysis. Examination of the two principal eigenvectors reveals broad overlap between individuals from these two language groups (Figure 3). There is no statistical difference in the position of these two groups along the principal eigenvectors (p = 0.45 and 0.22 for eigenvectors 1 and 2, respectively).

## Discussion

Populations in North Maluku have been substantially impacted by gene flow from Asia. At the ancestry informative loci tested here, we estimate the overall Asian admixture fraction to be ~67% (using two different methodologies). This fraction appears slightly higher on the X-chromosome, indicating a possible Asian female bias during the admixture process. In a regional context, these results mirror previously surveyed populations in eastern Indonesia. Several islands have broadly similar admixture profiles (*e.g.*, Sumba, Alor and Flores), which also show a trend toward female-biased Asian admixture using the same AIMs we screened here [11]. It is interesting to note that Asian admixture estimates for Sulawesi, which is the major island immediately to the west of North Maluku, are much higher than observed in the Maluku islands (97% among three Sulawesi populations). Indeed, from Sulawesi west, all Island Southeast Asian populations exhibit near-complete Asian ancestry at these genetic markers, while populations surveyed to the east of North Maluku (including coastal New Guinea) show a substantially lower Asian contribution. As such, the Molucca Sea, between Sulawesi and North Maluku, represents a geographic breakpoint, to the east of which chromosomes of Papuan ancestry have been retained at appreciable frequency. Interestingly, this phylogeographic pattern is mirrored by the distribution of Austronesian languages. West of the Molucca Sea, indigenous languages have been completely replaced by Austronesian languages; to the east of the Molucca Sea, indigenous languages have tended more often to persist [5,13]. Analyses of multiple genetic marker systems suggest a similar genetic breakpoint in the Nusa Tenggara region of Indonesia [11,14], an area that also marks a linguistic boundary between Papuan and Austronesian languages [5]. These patterns suggest that there is a major east-west divide in Asian influence, with respect to both genes and language, in Island Southeast Asia that runs from the Molucca Sea south to Nusa Tenggara. One possible explanation for this pattern is that speakers of Austronesian languages, carrying the Asian alleles at the AIMs we tested here, became less likely to replace existing populations (genetically or linguistically) as they moved east through Island Southeast Asia. Another alternative is that Papuan back-migration occurred subsequent to the Austronesian expansion, reaching its limits at the observed breakpoints. Both of these processes appear to be ongoing today [15].

It is remarkable that the genetic divide we observe at the Molucca Sea (and which has been observed previously in Nusa Tenggara) precisely follows a breakpoint in human morphological phenotypes first observed by the nineteenth century biologist Alfred R. Wallace [16]. Wallace described this phenotypic boundary as the line dividing people with Malay versus Papuan physical characteristics. This breakpoint is displaced to the east of his more famous biogeographical divide (*i.e.*, Wallace's Line), which he described based on the region's non-human fauna. Our data suggest that differing rates of Asian admixture, likely mediated by the geographical expansion of Austronesian-speakers, may be responsible for Wallace's phenotypic boundary observed for human populations in the region. Within North Maluku, Wallace also noted that individuals displayed what he deemed to be a composite of Papuan and Malay characteristics [16]. Similar observations have been reiterated by modern anthropologists [17-19]. The results we report here suggest that these observations may reflect genuine genetic admixture between Asian and Papuan populations. This diverse ancestry of individuals in North Maluku suggests that they may harbour more deeply divergent genetic lineages than might be expected for small island populations, which likely has implications for health and medical care in this area. In particular, a number of alleles that confer protection against severe malaria, and also sometimes have negative pleiotropic effects, have been associated with either Austronesian or Melanesian origins [20-25]. Given the lack of population structure that we observe, these functional genetic variants of diverse evolutionary origins may now be geographically widespread across North Maluku.

Despite the relatively strong contribution of Asian genetic material to the peoples of North Maluku, we observe no correlation between genes and language among our surveyed populations. Whether Papuan- or Austronesian-speaking, all populations shared similar levels of Asian admixture and showed no evidence of genetic differentiation. This result is not easy to reconcile with either the Replacement or Adoption Models. Unless swamped by subsequent gene flow, the Replacement hypothesis predicts some degree of genetic differentiation between populations speaking Austronesian versus Papuan languages. Likewise, the Adoption hypothesis predicts little genetic evidence of Austronesian admixture. Neither of these patterns is consistent with our observations. Instead, our results suggest a process of extensive Asian admixture with heterogeneous linguistic replacement. Austronesian languages made inroads in North Maluku, but this process only sporadically resulted in replacement of indigenous languages (predominantly on the southern and eastern coasts of Halmahera). Why some Papuan languages persisted in the face of widespread gene flow, and others did not, represents an intriguing open question.

This outcome contrasts strongly with other nearby island systems, including many populations in coastal Melanesia, where replacement of indigenous languages with Austronesian ones is complete, despite the persistence of Papuan genetic variation [10,26]. Indeed, within Island Southeast Asia, previous studies have found a heterogeneous relationship between genes and languages in different settings. For instance, Lansing et al. [27] found that on the island of Sumba there is a positive correlation between villages that have retained linguistic elements that likely trace to the single founding Austronesian language of the island and the frequency of Austronesian Y chromosome lineages. In contrast, Mona *et al*. [28] found no relationship between speakers of Austronesian versus Papuan languages with respect to Y chromosome or mtDNA variation in a multi-island comparison in the Indonesian province of East Nusa Tenggara. The island of New Guinea has produced especially mixed results suggestive of substantial local-scale heterogeneity in gene-language correlations; neither mtDNA or Y chromosome show associations with language in Indonesian West New Guinea, while studies of classical markers have sometimes shown a very strong association and sometimes not [29-32]. Regardless of the mechanism by which Austronesian languages replaced Papuan ones in North Maluku, our study suggests that deeply divergent languages do not necessarily represent a barrier to gene flow in this region, a pattern that is mirrored in many other parts of eastern Indonesia and the wider Melanesian area [33].

One important caveat of our AIM-based analysis is that it is designed expressly to tease out patterns of population genetic variation that have been shaped by Asian introgression into predominantly Papuan regions. Our finding of a lack of differentiation could be caused by either high rates of gene flow among populations or uniformly high rates of Asian admixture into individual populations, but not necessarily both. In other words, it would be premature to infer from our results that there exists no genetic structure that differentiates populations within North Maluku. However, if such population structure exists, it has not been caused by differential rates of Asian admixture. Other genetic systems may reveal important patterns of subdivision that result from alternative historical processes [34]. For instance, North Maluku was the major commercial hub for the long-distance spice trade to Asia and Europe during the last two millennia [35]. The genetic legacy of this global interaction may be better addressed using a different suite of genetic markers.

## Conclusions

Populations in North Maluku inhabit a remarkable linguistic contact zone reflecting deeply divergent Papuan and Austronesian languages. Our data suggest that the arrival of Austronesian languages was accompanied by extensive genetic admixture across North Maluku together with sporadic replacement of indigenous Papuan languages by Austronesian ones. In the broader context of the Austronesian expansion into this region, we have identified the Molucca Sea as an important phylogeographic and linguistic divide, east of which indigenous languages and genes resisted replacement more effectively than to the west. The cause of this pattern remains unclear, but likely reflects a geographical or social transition point in the expansion dynamics of the Austronesian dispersal [36].

## Methods

### Sample Collection

DNA samples were collected from unrelated adults of both sexes attending secondary school in the city of Ternate, North Maluku. Data on natal language and self-identified ethnicity were obtained from each individual. Because DNA collection occurred at a centralized location, the number of samples representing each population varied widely. For population-based analyses, we included in this study only those for which the sample size was at least five individuals. All samples were collected with informed consent by staff of the Eijkman Institute for Molecular Biology, using protocols approved by the Institute.

### AIM Genotyping

A total of 28 ancestry informative markers (AIMs) (see Additional File 1) were selected from previous work [11]. Briefly, these AIMs were identified using a SNP screening process designed to find those that showed high F_ST _between southern Han Chinese and PNG highlanders. Candidate markers were chosen from two sources: the HOMINID dataset, a collection of re-sequenced putatively neutral regions distributed across the human genome [37], and the Jakobsson et al. dataset, a collection of 500,000 SNPs typed in the HGDP-CEPH panel [38]. To address the question of sex-specific admixture, markers were selected from both the autosomes and the X chromosome. To minimize the effects of natural selection, all AIMs were located away from genes (including introns, UTRs and immediate flanking regions) and are more than 1 cM distant from other markers in the panel (*i.e.*, they are evolutionarily and statistically independent).

AIM genotype data were collected using a MassARRAY^® ^iPLEX Gold SNP-typing platform (Sequenom) at the University of Arizona Genetics Core facility (Additional File 3). Genotype data from the 28 AIMs were initially checked for agreement with Hardy-Weinberg proportions and evidence of minimal sample dropout. One autosomal marker, rs12613102, showed significant deviations from expected Hard-Weinberg proportions and a high number of samples produced no genotype calls. The affected samples were not associated with missing data at other markers, and this marker was eliminated from all further analyses (data are not shown in Additional File 1).

### Data Analysis

Admixture estimates and confidence intervals were generated for each population using a variant of the admixture estimation method introduced by Chakraborty et al. [39], as modified by Cox et al. [11]. Briefly, we inferred admixture rates using a weighted least-squares estimator [39] altered to account for the sampling error in each of the 'parental' (P1 and P2) and 'hybrid' (H) populations by inferring a frequency density for P1, P2 and H at each marker. Using a re-sampling approach, random variables were drawn from the three frequency distributions, admixture was calculated using the least-squares estimator and the process was repeated 10^5 ^times. The median admixture rate with 95% confidence intervals (*i.e.*, 0.025 and 0.975 quantiles) was calculated from the distribution of re-sampled admixture rates. Note that this approach explicitly accounts for variation in sample sizes and effective population sizes, even when they are quite small and/or differ widely between populations.

Population differentiation was assessed using several methods. First, we employed a hierarchical analysis of variance (AMOVA), as described by Weir [40]. For this analysis, we considered populations, which were determined by self-identified ethnicity (described above), as a subordinate classification to language group (Austronesian- versus Papuan-speakers). Language group affiliations were determined using data compiled in [5]. Significance of individual components of variance (*i.e.*, F_ST_, F_CT _and F_SC_) was assessed using 1,000 random permutations of the data. We implemented the AMOVA using the program Arlequin version 3.5.1.2 [41]. Our second examination of differentiation among populations used the Bayesian clustering algorithm STRUCTURE (v. 2.3.3) [42]. We implemented STRUCTURE using a dataset containing AIM genotypes from our test samples from North Maluku together with the "parental" populations from which our ancestry informative markers were initially chosen (southern Han Chinese and highland Papua New Guinea). Data for the parental populations was taken from [11]. We ran STRUCTURE with a burn-in period of at least 45,000 steps and 5 × 10^6 ^steps per run, varying K (the number of populations) from 1 to 5. We ran each condition multiple times to ensure similarity of results among runs. To improve the detection of structure in our data we allowed prior information about individuals to inform the clustering process, as described by Hubisz et al. [43]. All results shown here use language affiliation (*i.e*., Papuan versus Austronesian) as prior information. Use of ethnicity produced very similar results (data not shown).

As a final examination of population structure, we performed an individual-based principal components analysis (PCA), implemented using the program SMARTPCA [44], to test for differentiation between Austronesian- and Papuan-speakers. In addition to providing a graphical depiction of the position of each individual along the principal eigenvectors, this method applies a formal statistical test based on Tracy-Widom theory to detect structure in the dataset.

## Authors' contributions

JAW conceived of the study, assisted with data collection, performed data analysis, and drafted the manuscript. MPC performed data analysis, statistics and edited the manuscript. AWS, AP and RA assisted with data collection. AH and HS helped conceive of the study and oversaw sample collection. All authors have read and approved the final manuscript.

## Supplementary Material

Additional file 1 **AIM frequencies for all samples (Spreadsheet)**. Click here for file

Additional file 2 **Hierarchical analysis of population differentiation by individual ancestry informative marker (Spreadsheet)**. Click here for file

Additional file 3 **SNP genotyping primer details and reaction conditions (Spreadsheet)**. Click here for file
